# Policy-driven multidisciplinary primary care after stroke or Transient Ischaemic Attack (TIA) improves survival – an observational target trial evaluation involving linked registry data

**DOI:** 10.23889/ijpds.v9i5.2638

**Published:** 2024-09-18

**Authors:** Nicole Satherley, Andrew Sporle

**Affiliations:** 1 iNZight Analytics; 2 University of Auckland

## Objective

To determine the effectiveness of government policies supporting coordinated multidisciplinary primary care (MDC) in improving long-term survival following stroke or Transient Ischaemic Attack (TIA).

## Approach

We used the target trial framework for observational data to assess the average population effect of primary care MDC policies. The cohort comprised patients from the Australian Stroke Clinical Registry (January 2012-June 2015) linked with (i) Australian Medicare claims to define exposures (MDC claims in the 6-18 months post-stroke); (ii) hospital, pharmaceutical and aged care datasets for additional covariates; (iii) National Death Registry for survival outcomes (19-30 months post-stroke). Level of impairment was classified by latent class analysis using EQ-5D-3L questionnaire data obtained 90-180 days post-stroke. Multilevel survival analysis with inverse probability treatment weights was applied.

## Results

Among 7,255 people with stroke (42% female, median age 71 years, 24% TIA, level of impairment: 39% minimal, 32% moderate, 29% severe), 29% had a Medicare claim for MDC (23% minimal, 31% moderate, 39% severe). Mortality was reduced in those receiving a claim (vs non-receipt) in the minimal (adjusted Hazard Ratio (aHR): 0.50, 95%CI:0.27, 0.91) and severe (aHR: 0.65, 95%CI:0.46, 0.91) impairment groups, but not the moderate impairment group (aHR: 1.31, 95%CI:0.86, 1.99). Group differences in allied health services claimed during MDC were observed: secondary prevention (14% minimal vs 10% severe impairment), rehabilitation (21% minimal vs 25% severe impairment).

## Implications

Drawing causal inferences from linked observational data demonstrated the population effectiveness of primary care MDC policies, in improving survival following stroke/TIA, with variation by impairment class.

